# Brain Connectivity Changes in Autosomal Recessive Parkinson Disease: A Model for the Sporadic Form

**DOI:** 10.1371/journal.pone.0163980

**Published:** 2016-10-27

**Authors:** Elena Makovac, Mara Cercignani, Laura Serra, Mario Torso, Barbara Spanò, Simona Petrucci, Lucia Ricciardi, Monia Ginevrino, Carlo Caltagirone, Anna Rita Bentivoglio, Enza Maria Valente, Marco Bozzali

**Affiliations:** 1 Neuroimaging Laboratory, IRCCS Santa Lucia Foundation, Rome, Italy; 2 Clinical Imaging Sciences Centre, Brighton & Sussex Medical School, Falmer, Brighton, United Kingdom; 3 IRCCS Casa Sollievo della Sofferenza, CSS-Mendel laboratory, San Giovanni Rotondo, Italy; 4 Dept. of Neurology and Psychiatry, Sapienza University of Rome, Rome, Italy; 5 Sobell Dept. of Motor Neuroscience and Movement Disorders, Institute of Neurology, University College London, London, United Kingdom; 6 Dept. of Clinical and Behavioural Neurology, IRCCS Santa Lucia Foundation, Rome, Italy; 7 Dept. of Neuroscience, University of Rome ‘Tor Vergata’, Rome, Italy; 8 Dept. of Neurosciences, Catholic University, Rome, Italy; 9 Section of Neurosciences, Dept. of Medicine and Surgery, University of Salerno, Salerno, Italy; University of Manchester, UNITED KINGDOM

## Abstract

Biallelic genetic mutations in the *Park2* and *PINK1* genes are frequent causes of autosomal recessive PD. Carriers of single heterozygous mutations may manifest subtle signs of disease, thus providing a unique model of preclinical PD. One emerging hypothesis suggests that non-motor symptom of PD, such as cognitive impairment may be due to a distributed functional disruption of various neuronal circuits. Using resting-state functional MRI (RS-fMRI), we tested the hypothesis that abnormal connectivity within and between brain networks may account for the patients’ cognitive status. Eight homozygous and 12 heterozygous carriers of either *PINK1* or *Park2* mutation and 22 healthy controls underwent RS-fMRI and cognitive assessment. RS-fMRI data underwent independent component analysis to identify five networks of interest: default-mode network, salience network, executive network, right and left fronto-parietal networks. Functional connectivity within and between each network was assessed and compared between groups. All mutation carriers were cognitively impaired, with the homozygous group reporting a more prominent impairment in visuo-spatial working memory. Changes in functional connectivity were evident within all networks between homozygous carriers and controls. Also heterozygotes reported areas of reduced connectivity when compared to controls within two networks. Additionally, increased inter-network connectivity was observed in both groups of mutation carriers, which correlated with their spatial working memory performance, and could thus be interpreted as compensatory. We conclude that both homozygous and heterozygous carriers exhibit pathophysiological changes unveiled by RS-fMRI, which can account for the presence/severity of cognitive symptoms.

## Introduction

Parkinson’s disease (PD) is the second most common neurodegenerative disorder after Alzheimer’s disease in the population aged over 65 years. The core features of PD include resting-tremor, rigidity, bradykinesia and postural instability, but non-motor symptoms such as cognitive decline, neuropsychiatric disorders and dysautonomia are also frequently observed [1]. Relevant for clinical management, cognitive deficits can be present in PD since the early clinical stages, including frontal-executive dysfunction, difficulties with set-shifting, visuospatial deficits, and impairments in learning and memory [2]. In recent years, researchers have put increasing efforts to clarify the pathophysiology of cognitive impairment in PD and, to this aim, the availability of a condition mimicking preclinical stages in humans is of great interest. Relevant progress in this field has come from studies of mendelian forms of parkinsonism, in particular those recessively inherited. Biallelic mutations in three genes (*Park2*/Parkin, *PINK1*, and less frequently *DJ-1*) are mainly responsible for a fully penetrant, autosomal recessive PD phenotype (ARPD). ARPD is clinically characterized by early onset, slow progression, excellent response to levodopa, and variable occurrence of additional features such as dystonia at onset, sleep benefit, hyperreflexia and psychiatric symptoms [3]. Interestingly, single heterozygous mutations in *Park2* and *PINK1* genes can be identified in patients with features indistinguishable from sporadic, late-onset PD, as well as in non-symptomatic individuals [4]. These mutations are regarded as minor susceptibility factors modulating the risk for developing PD in a multifactorial context. Moreover, there is growing evidence indicating that even non-symptomatic heterozygous carriers (i.e., relatives of patients with biallelic mutations) often present with subtle signs of dopaminergic dysfunction, as demonstrated by Photon Emission Tomography and functional MR imaging (fMRI) [5,6]. These individuals provide therefore a unique model for *in vivo* research into the pre-clinical stages of PD. Additionally, it was shown that carriers of single mutations in *PINK1* or *Park2* genes show a similar phenotype at a brain network level [7], which is consistent with the closely related dysfunctional effect of gene disruption in several *in vitro* and *in vivo* models [8]. To date, a few studies investigating the cognitive profile of individuals with ARPD have been published. Three of them have consistently reported that *Park2*-mutated patients perform similarly or even better than non-mutated patients on cognitive testing [9,10]. On the other hand, other studies have described cognitive impairment in patients with *Park2* mutations [11], as well as non-specific cognitive deficits in healthy heterozygous carriers [10]. We recently published neuropsychological data from *PINK1* homozygous and heterozygous mutation carriers followed-up for 12 years, based on the Montreal Cognitive Assessment battery (MoCA) and an extensive battery exploring all principal cognitive domains [12]. Interestingly, all affected homozygotes and 5 out of 14 heterozygotes reported abnormal scores at the MoCA and at tests sensitive to frontal dysfunction, consistently with the dysexecutive syndrome which is typically observed in sporadic PD [13]. The pathophysiological basis of these non-motor manifestations cannot be completely attributed to dysfunction of the basal ganglia, and may be the consequence of distributed functional disruption in various neuronal circuits [14]. Against this background, functional imaging studies may provide relevant insights, especially in the absence of macroscopic brain abnormalities. Resting-state fMRI (RS-fMRI) has gained particular value for the investigation of cognitive symptoms in neurodegenerative diseases. This non-invasive MRI technique relies on the neural spontaneous blood-oxygen-level dependent (BOLD) signal fluctuations to estimate the intrinsic activity synchronization across the entire brain at rest, without requiring any experimental task [15]. So far, distinct RS-fMRI networks have been identified in healthy subjects [15], and selective disruptions in their functional connectivity (FC) have been observed in various neurodegenerative disorders including sporadic PD [16]. In particular, the default-mode network (DMN), whose disruption has been associated to impairment of global cognition [17], was recently investigated in patients with PD, showing reduced FC in the medial temporal lobe and in the inferior parietal cortex [18]. Other networks are also likely to be implicated in PD, and might exhibit distinct patterns of abnormalities at different disease stages. For instance, in a genetic variant of frontotemporal dementia, FC was shown to play in distinct networks either a pathogenetic or a compensatory role when assessed at preclinical or clinical stages of disease [19].

A key question in PD is to determine whether different RS-fMRI networks interact with each other in determining higher level functions and dysfunctions across disease evolution (i.e., inter-network connectivity) [20]. The current study aims at investigating the role of dynamic changes across five major networks of interest (i.e., DMN, salience network [SN], executive network [ExN], right and left fronto-parietal networks [rFP] and [lFP]) in determining the cognitive status of individuals with different mutational loads in subjects with ARPD-causative genes. To this purpose, we recruited homozygous (HOM) and heterozygous (HET) carriers of either *PINK1* or *Park2* mutations. In the frame of this experimental model, we attempted to clarify the pathophysiology of cognitive impairment in PD when moving from preclinical, or very early (HET individuals) to overt disease stages (HOM individuals). From a clinical/neuropsychological viewpoint, our prediction was to identify visuospatial memory deficits, which are typical of PD, in HOM individuals. Conversely, in HET individuals, we expected to identify preclinical FC modifications in the absence of obvious neuropsychological deficits.

## Materials and Methods

### Participants

Eight HOM patients (5 *PINK1* and 3 *Park2* mutation carriers; M/F = 6/3; mean age = 51.4, SD = 8.1 years), 12 HET relatives (10 *PINK1* and 2 *Park2* mutation carriers; M/F = 5/10; mean age = 40.2, SD = 14.7 years) and 22 age- and gender-matched healthy controls (HC; M/F = 10/12; mean age = 47.0, SD = 12.3 years) took part in the study (see Table 1 for demographic, clinical and pharmacological characteristics). The diagnosis of clinically definite or probable PD was made according to the clinical diagnostic criteria of the UK PD Society Brain Bank [21], with the only exception that positive family history was not considered as an exclusion criterion. Disease severity was estimated by the Hoehn & Yahr stages and the Unified PD Rating Scale [22, 23]. Major systemic, psychiatric, and other neurological illnesses were carefully investigated and excluded in all subjects. Local Ethical Committee approved the project and written informed consent was obtained by all participants before study initiation.

**Table 1:** Principal demographic and clinical characteristics of studied subjects. Abbreviations: HET = heterozygous; HOM = homozygous; HC = healthy controls; SD = standard deviation; LEDD = Levodopa equivalent daily dose; UPDRS = Unified Parkinson's Disease Rating Scale; MOCA = Montreal Cognitive Assessment; H&Y = Hoehn and Yahr scale.

	HC (n = 22)	HET (n = 12)	HOM (n = 8)
**Age at scan (SD)**	47.0 (12.2)	41.9 (14.8)	51.4 (8.1)
**Sex (M/F)**	10/12	5/7	6/2
**Educational level (SD)**	14.7 (3.2)	12.3 (2.1)	11.4 (2.3)*
**MOCA**	/	23.8 (1.8) °	23.0 (2.6) °
**Diagnosis**	/	10 unaffected/ 2 possibly affected	Affected
**Disease duration**	/	/	18.2 (8.5)
**Pink/Parkin**	/	10/2	5/3
**LEDD**	/	/	590.9 (358.1)
**UPDRS**	/	1.7 (3.7)	23.2 (19.9)#
**H&Y**	/	/	2.2 (0.9)

* HC vs. HOM/HET p< 0.05

° pathological score

^#^ HOM vs. HET p< 0.05.

### Neuropsychological assessment

Cognitive assessment was performed by two trained neuropsychologists on the same day of MRI acquisition. The MoCA [24] and the Frontal Assessment Battery (FAB) [25] were administered to all subjects. Additionally, mutation carriers underwent the following battery of tests: 1) Verbal episodic long-term memory: Immediate and Delayed recall of a 15-Word List [26]; Short Story Recall [27]; 2) Visuo-spatial episodic long-term memory: Delayed recall of Complex Rey’s Figure [28]; 3) short-term memory: Digit-span and Corsi Block Tapping task [29]; 4) Executive functions: Phonological Word Fluency [26]; Categorical Word Fluency [27]; Trail Making Test [30]; Stroop test [31]; 4) Problem-solving: Raven’s Colored Progressive Matrices [26]; Praxis: Copy of drawings [27]; Copy of Complex Rey’s Figure [28]. For each test, appropriate adjustments for gender, age, and education were applied according to the Italian normative data. In addition, available cut-off scores of normality (95% of the lowest tolerance limit of the normal population distribution) were applied.

Subjects were considered as “cognitively impaired” if they reported pathological scores at MoCA [3]. Scores obtained at other tests were used to assess group differences between HOM and HET individuals and for correlations with imaging data. In the former case, a series of t-tests for independent samples were used (statistical threshold = p<0.003 after Bonferroni’s correction).

### MRI

All subjects underwent MRI at 3T (Magnetom Allegra, Siemens, Erlangen, Germany), including the following acquisitions: 1) Dual-echo turbo spin-echo (TSE) (TR = 6.190 ms, TE = 12/109 ms); 2) fast-FLAIR (TR = 8.170 ms, TE = 96 ms, TI = 2.100 ms); 3) 3D Modified-Driven-Equilibrium-Fourier-Transform (MDEFT) scan (TR = 1338 ms, TE = 2.4 ms, Matrix = 256x224x176, FOV = 250x250 mm^2^, slice thickness = 1 mm); 4) T2*weighted echo-planar image (EPI) sensitized to BOLD contrast (TR = 2080 ms, TE = 30 ms, 32 axial slices parallel to AC-PC line, matrix: 64x64, pixel size = 3x3 mm^2^, slice thickness = 2.5 mm, flip-angle = 70°) for RS-fMRI. BOLD EPIs were collected during rest for 7 min and 20s, resulting in a total of 220 volumes.

### Image analysis

Dual-echo TSE and FLAIR images were reviewed by a neurologist expert in MRI to assess/exclude the presence of macroscopic abnormalities.

For each subject the first four volumes of the RS-fMRI series were discarded to allow for T1 equilibration effects. Statistical parametric mapping (SPM8; www.fil.ion.ucl.ac.uk\spm) was used for image preprocessing and statistical comparison of RS-fMRI data. The preprocessing steps included correction for head motion (using the standard realignment algorithm in SPM8), compensation for slice-dependent time shifts, and co-registration with the corresponding MDEFT. The MDEFT was segmented using the segmentation algorithm in SPM8, and the resulting grey matter (GM) images were used to compute every subject’s total GM volume. The segmentation also provides the normalization parameters that map the subject’s brain into Montreal Neurological Institute coordinates. The same parameters were applied to the motion and slice-timing corrected EPI images. Then, they were filtered by a phase-insensitive band-pass (0.01–0.08 Hz) to reduce the effect of low frequency drift and high frequency physiological noise. Finally, smoothing with a 3D-Gaussian Kernel of 8 mm^3^ FWHM was applied. Group Independent Component Analysis (ICA) fMRI Toolbox (GIFT, www.icatb.sourceforge.net) was used for component decomposition and set to identify 20 independent components. Results were converted to Z-scores. The components were reviewed to identify the DMN, the SN, the ExN, the lFP and the rFP networks [15].

### Intra-network analysis

To statistically evaluate intra-network FC of each selected network, second level analyses were implemented in SPM8 on participants’ reconstructed spatial maps. First, we performed a cross-sectional analysis (with a full-factorial design) with the group of HC, HET and HOM as main factor and GM volume and years of education as covariates of no interest, for each single network separately. Then, a correlation analysis was performed using a two sample T-test, with group belonging (HOM or HET) as factor, the Corsi score as covariate of interest, and the total GM volume and type of genetic mutation (*PINK1/Park2*) as covariates of no interest. Results were accepted as significant at p<0.05 FWE cluster-level corrected.

### Inter-network analysis

To statistically evaluate inter-network FC, subject specific network time courses were detrended and pairwise correlated by Pearson's correlation, following an established procedure [32,33]. Briefly, we computed the constrained maximal lagged correlation between all pair-wise combinations of networks. Correlation coefficients and corresponding p values in each pair of networks were calculated for different lags (ranging from -12 to 12), where lags were circularly shifted. The best p value (corresponding to the optimal lag between two networks) was used in the subsequent analysis. To assess between-group differences, correlation coefficients were transformed to z-scores using the Fisher's z-transformation and entered into a between-subject ANOVA with education and total GM volumes as variables of no interest (p<0.05, Bonferroni-corrected).

Finally, in order to investigate the association between inter-network connectivity and severity of cognitive symptoms, we correlated the internetwork z-scores of each pair of networks with cognitive scores, with the total GM volume as covariate of no interest.

## Results

### Clinical and neuropsychological evaluation

There was a statistically significant difference in the average number of years of education between HC and HOM, but not between HET and either HOM or HC. The years of education were subsequently introduced as a covariate of no interest in all fMRI analyses. All other demographic features were matched across groups (Table 1).

All HOM patients had a diagnosis of clinically definite PD, as confirmed by the Unified PD Rating Scale (Table 1). In the HET group, two *PINK1* carriers received a diagnosis of possible PD (subjects F1-IV:13/M and F1-IV:12/M, already reported in a previous publication) [12].

With respect to cognitive assessment (Table 2), all but two PD individuals (one from the HOM, one from the HET group; carriers of PINK1 mutation in both cases) reported pathological scores at MoCA, and were classified as cognitively impaired. Interestingly, all *Park2* mutation carriers (3 HOM and 2 HET) showed cognitive impairment. When comparing HOM and HET subjects for their performance in single cognitive domains, the former group resulted significantly more impaired in visuo-spatial working memory.

**Table 2:** Performance scores obtained by HET and HOM individuals on neuropsychological tests. In brackets cut-off for normative values; in bold characters pathological values. Abbreviations: Edu: educational level; HET: heterozygous; HOM: homozygous; MOCA: Montreal cognitive assessment; LTM: Long term memory; STM: Short term memory; Imm: Immediate recall; Del: delayed recall; RMP47: Raven’s Progressive Matrices; MCST: Modified Card Sorting Test- criteria.

				LTM: Verbal 15-words list		LTM: Visuo-spatial Complex Rey's Figure		STM		Reasoning	Constructional praxis			Executive functions	
			MOCA	Imm	Del	Imm	Del	Digit span	Corsi span	RMP47	Copy of drawings	Copy of drawings with landmarks	Copy of Complex Rey's Figure	Phonological Word Fluency	MCST criteria
Group	Mutation	Edu	*(>26)*	*(>28*.*5)*	*(>4*.*6)*	*(>6*.*4)*	*(>6*.*3)*	*(>3*.*7)*	*(>3*.*5)*	*(>18*.*9)*	*(>7*.*1)*	*(>61*.*8)*	*(>23*.*7)*	*(>17*.*3)*	*(>4*.*2)*
***Heterozygous***															
HET1	*Parkin*	8	**21.0**	45.3	7.7			4.8	3.5	**16.4**	8.8	67.4	**8.2**	39.9	**1.0**
HET2	*Parkin*	13	**23.0**	47.7	11.4	28.0	26.8	4.6	4.9	32.2	11.5	69.2	34.2	31.7	6.0
HET3	*Pink1*	13	**24.0**	36.8	6.1	12.7	18.4	6.8	4.7	27.8	11.2	68.8	34.4	28.5	6.0
HET4	*Pink1*	13	**22.0**	40.5	6.0	**5.9**	6.3	5.5	4.5	22.8	10.2	68.7	34.3	41.4	5.0
HET5	*Pink1*	13	**24.1**	30.6	6.7	11.0	13.0	**2.6**	4.9	28.5	9.6	67.4	33.4	**12.0**	6.0
HET6	*Pink1*	8	**24.1**						4.6						
HET7	*Pink1*	13	**24.0**	**19.3**	**2.2**	**1.2**	**2.1**	4.9	4.4	26.0	10.0	68.5	26.9	20.8	6.0
HET8	*Pink1*	13	28.0	31.0	4.5	**5.1**	9.5	5.8	4.5	27.2	11.1	68.6	34.0	**8.0**	5.0
HET9	*Pink1*	11	**24.1**						4.6						
HET10	*Pink1*	13	**23.0**	**25.6**	4.3	**5.5**	**5.2**	5.9	4.6	23.1	11.0	68.5	30.9	24.9	6.0
HET11	*Pink1*	8	**23.0**	**18.8**	**1.8**	17.5	13.3	4.3	3.7	26.5	8.7	**59.4**	30.7	**11.9**	6.0
HET12	*Pink1*	15	**26.0**	**11.3**	**0.3**	15.8	16.1	6.7	5.4	27.5	10.8	68.3	29.4	25.9	6.0
***Homozygous***															
HOM1	*Parkin*	13	**23.0**	44.6	6.7	8.6	9.1	4.7	3.6	26.5	7.3	64.0	32.8	34.0	6.0
HOM2	*Parkin*	13	**24.0**	38.1	9.2	19.0	15.5	5.6	3.7	32.0	10.4	67.2	35.1	29.5	6.0
HOM3	*Parkin*	13	**21.0**	27.8	8.1	9.7	**3.9**	4.8	4.7	25.8	11.2	68.8	25.9	17.5	6.0
HOM4	*Pink1*	13	**19.0**	**22.9**	**3.5**	**4.5**	**2.6**	4.5	3.9	**13.3**	**5.7**	**49.7**	**6.0**	**12.8**	6.0
HOM5	*Pink1*	8	28.0	26.3	6.0	9.4	**5.8**	4.1	3.8	**17.6**	9.0	64.8	31.4	**14.9**	
HOM6	*Pink1*	8	**23.0**	41.3	7.7	17.4	17.5	5.0	4.1	31.4	11.4	68.5	34.5	30.5	6.0
HOM7	*Pink1*	10	**23.0**	34.3	6.5	19.6	20.6	5.9	3.8	30.3	10.8	69.7	34.1	35.0	6.0
HOM8	*Pink1*	13	**23.0**	**20.7**	5.4	6.5	14.3	4.6	3.9	28.2	9.5	68.2	34.2	19.7	6.0

### Intra-network RS-fMRI

**Fig 1:**
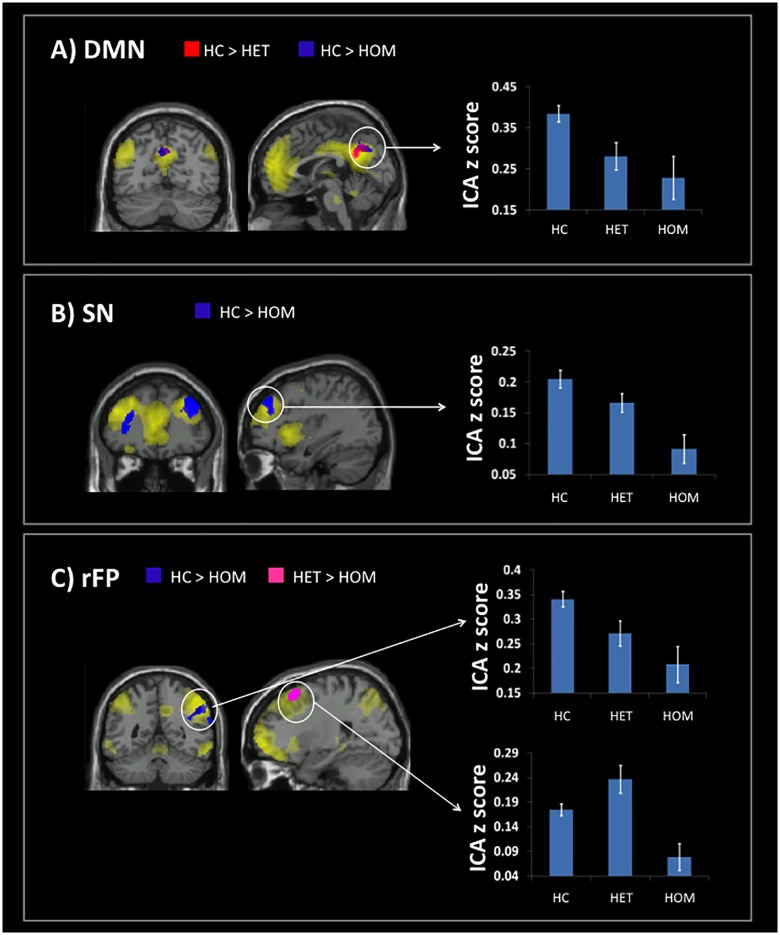
Between group FC changes within the RS-fMRI networks of interest. This figure illustrates between-group differences in functional brain connectivity observed in individual networks: A) the default mode network (DMN), B) the executive network (ExN), and C) the right fronto-parietal network (rFP). In all cases, the networks (main effect of groups) are shown in yellow. Red areas show the regional pattern of reduced connectivity in homozygous mutation carriers (HOM) as compared to healthy controls (HC). Blue areas show the regional pattern of reduced connectivity in heterozygous mutation carriers (HET) as compared to HC. Pink areas show the regional pattern of reduced connectivity in HOM as compared to HET. For each contrast, the signal plots on the right show the group level of connectivity at the peak of some clusters.

All RS networks of interest were detectable from ICA decomposition. As expected, the DMN included the posterior and anterior cingulate cortex and the right and left inferior parietal nodes; the rFP and lFP networks included the right/left anterior insula, the medial prefrontal cortex, and the right/left frontal and parietal regions; the ExN included the dorsolateral prefrontal and the posterior parietal cortex; the SN included the hippocampus, parahippocampal gyrus, retrosplenial cortex, posterior cingulate cortex, precuneus, temporo-parietal junction, angular gyrus, lateral temporal cortex, ventrolateral prefrontal cortex, and medial prefrontal cortex. Between-group comparisons of intra-network connectivity are shown in Table 3 and Fig 1.

**Table 3:** Changes in FC in five networks of interest. Brain areas of significant FC alteration in RS-networks (A) and of significant correlation between Corsi score and FC (B) HOM ARPD.

				MNI coordinates				
	Brain area	Size	R/L	x	y	z	T-value	p value
(A)								
	**Default mode network**							
	**HC > HET**							
	Posterior cuyngulate gyrus/Precuneus	558	L	-2	-52	28	5.01	0.002
	**HC > HOM**							
	Precuneus	413	L	-4	-64	36	5.15	0.01
	**Executive network**							
	**HC > HOM**							
	Frontal pole	517	R	26	48	26	4.82	0.002
	Frontal pole	298	L	-26	46	26	4.25	0.05
	**Right working memory network**							
	**HC > HOM**							
	Angular gyrus/Supramarginal gyrus	472	R	62	-60	20	4.40	0.005
	**HET > HOM**							
	Superior frontal gyrus	456	R	20	26	58	4.38	0.05
(B)								
	**Right working memory network**							
	Superior frontal gyrus	731	R	18	32	44	8.63	0.000
	**Left working memory network**							
	Middle frontal gyrus	224	L	-32	4	48	9.12	0.03
	**Salience netwrork**							
	Paracyngulate gyrus	297	L	-10	38	28	6.09	0.031
	**Executive network**							
	Ant cingulate gyrus	266	R	4	0	34	5.35	0.005

Within the DMN, both HET and HOM subjects showed lower FC than HC in the precuneus. Within the ExN, HOM patients showed decreased connectivity than HC in the frontal pole bilaterally (a similar finding was detectable, at uncorrected level, also in HOM patients compared to HET individuals). Within the rFP network, HOM patients showed decreased FC in the right angular/supramarginal gyrus when compared to HC, and in the right superior frontal gyrus when compared to HET individuals.

**Fig 2:**
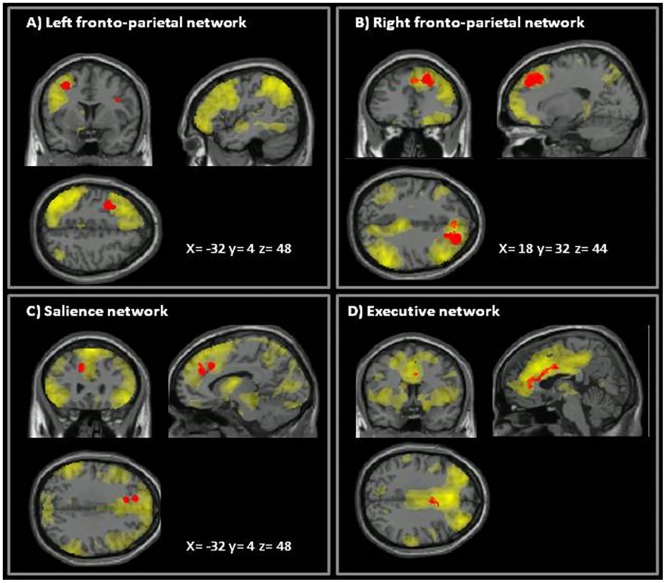
Associations between single network FC and spatial working memory performance in HOM patients. Within each network (shown in yellow), red areas illustrate the brain regions whose functional connectivity was positively associated with scores reported by homozygous (HOM) mutation carriers at the Corsi test (spatial working memory). These regions included: the left middle frontal gyrus within the left fronto-parietal network (A); the right superior frontal gyrus within the right fronto-parietal network (B); the left paracingulate gyrus within the salience network (C); and the right anterior cingulate gyrus within the executive network (D).

Positive correlations were found between the Corsi scores reported by HOM, but not by HET individuals, and their FC in various networks (Fig 2). Within the rFP, this pattern of correlation was found in the right superior frontal gyrus; within the lFP it was found in the left middle frontal gyrus; in the SN it was localized in the left paracingulate gyrus; and finally, in the ExN, it was localized to the anterior cingulate gyrus.

### Inter-network RS-fMRI

The inter-network FC correlation was evaluated for ten pairs of networks (resulting from all pair-wise combinations of 5 networks). Table 4 summarizes the optimal lag, r, and p vales of correlation for each pair of networks in each group (HC, HET, HOM).

**Table 4:** Inter-network correlation results. Mean lag, Pearson r index and p values of the inter-network FC correlations in the group of healthy controls (HC), HET individuals and HOM patients.

		*lag*	*r*	*p*
**DMN_ExN**	***HC***	-0.136	-0.451	0.029
	***HET***	0.214	-0.434	0.001
	***HOM***	-0.375	-0.355	0.031
**DMN_lFP**	***HC***	-0.273	-0.387	0.001
	***HET***	-2.286	0.319	0.016
	***HOM***	1.250	-0.351	0.005
**DMN_rFP**	***HC***	-0.682	-0.305	0.006
	***HET***	-0.643	0.332	0.002
	***HOM***	0.375	-0.476	0.000
**DMN_SN**	***HC***	-1.136	-0.370	0.006
	***HET***	-0.571	0.359	0.003
	***HOM***	1.000	-0.391	0.000
**lFP_ExN**	***HC***	-0.136	0.357	0.009
	***HET***	-0.429	-0.329	0.011
	***HOM***	0.750	0.269	0.059
**lFP_rFP**	***HC***	0.318	0.387	0.005
	***HET***	-0.643	0.458	0.000
	***HOM***	0.250	0.491	0.001
**rFP_ExN**	***HC***	1.500	0.311	0.027
	***HET***	-0.571	-0.364	0.004
	***HOM***	1.625	0.329	0.011
**SN_ExN**	***HC***	-0.591	0.384	0.005
	***HET***	0.000	0.395	0.001
	***HOM***	0.750	0.401	0.019
**SN_lFP**	***HC***	-0.318	0.392	0.011
	***HET***	-1.500	-0.273	0.058
	***HOM***	0.000	0.320	0.016
**SN_rFP**	***HC***	-0.273	0.335	0.005
	***HET***	-0.143	0.299	0.011
	***HOM***	-0.250	0.526	0.001

**Fig 3:**
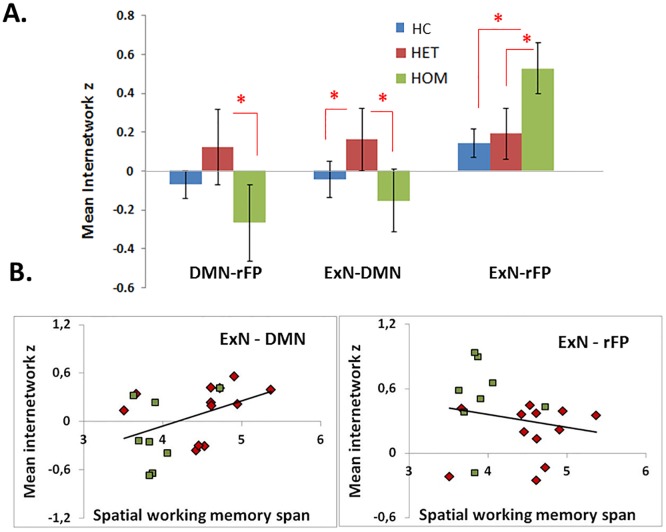
Inter-network analysis. Panel A shows the mean (SD) internetwork z scores for each studied group (i.e., healthy controls, HC; homozygous (HOM) and heterozygous (HET) mutation carriers) in three pairs of networks: salience network and right fronto-temporal network (SN-rFP); default mode network and right fronto-temporal network (DMN—rFP); salience network and default mode network (SN-DMN). Asterisks highlight significant between-group differences. Panel B illustrates the significant correlations, obtained in HOM (green-square marks) and HET (red-rhomb marks) mutation carriers altogether, between mean internetwork z scores in SN-rFP and SN-DMN pairs of networks and individual scores obtained at Corsi test (spatial working memory span).

Three significant differences in the z-values (expressing the strength of between-network correlation in FC) for patients versus controls and between the two genetic groups (HOM and HET) were identified using an ANOVA model with “group” as factor. As shown in Fig 3A, these group differences in inter-network connectivity were found between SN-rFP (F(1,42) = 3.87, p<0.01), DMN-rFP (F(1,42) = 4.10, p<0.01) and SN-DMN (F(1,42) = 3.88, p<0.01). In the case of SN-rFP, the difference was driven by an increased inter-network connectivity in the HOM group compared to both, HC (t(42) = 3.12, p<0.01) and HET individuals (t(42) = 3.04, p<0.01), while no significant difference was found between HC and HET individuals (t(42)<1). In the case of DMN-rFP, the significant main effect was driven by reduced inter-network FC correlation in HOM as compared to HET (t(42) = 2.47, p< 0.05) and an increased connectivity in HET as compared to HC (t(42) = 2.00, p< 0.058). Finally, the difference in the SN-DMN was driven by an increase of inter-network FC in the group of HET compared to both HC (t (42) = 2.12, p< 0.05) and HOM (t(42) = 2.37, p< 0.05), whereas no difference was found between HC and HOM individuals (t(42)<1).

The inter-network connectivity z scores were correlated with the performance at Corsi test. In the SN-rFP pair, z scores were negatively correlated with the Corsi scores across the two genetic groups (HOM, HET), indicating that an increase in the connectivity between these two networks (mainly present in the HOM group) was associated with a worse performance in visuo-spatial working memory.

The inter-network connectivity scores in the SN-DMN and DMN-SN pairs of networks correlated positively with the Corsi score (r = 0.49, p<0.04 and r = 0.48, p<0.04 respectively). An increased connectivity, mainly present in HET individuals, was associated with a better performance in visuo-spatial working memory, indicating possible compensating mechanisms. These correlations are shown in Fig 3B.

Levodopa treatment is known to potentially affect functional brain connectivity [34], and therefore constitutes a potential bias in this study, as only the HOM group is under treatment. To estimate the magnitude of this effect, we tested for correlations between individual levodopa equivalent daily dose (LEDD) and FC in all networks of interest in HOM patients (the only group under medication). Within the limitation of our small sample size we were unable to detect any significant association between LEDD and FC.

## Discussion

In this study, we recruited subjects carrying single or biallelic mutations in either *PINK1* or *Park2* which, theoretically, may be regarded as a model for preclinical and clinical stages of PD. Mutations in these two genes have been shown to result in a similar phenotype at a brain network level [7], allowing individuals with *PINK1* and *Park2* mutations to be included in the same experimental setting.

Consistently, all HOM patients responded to a diagnosis of clinically definite PD, while 10 out of 12 subjects from the HET group were classified as clinically unaffected. The focus of this study was to identify, using RS-fMRI, the pathophysiological substrates for the cognitive status of HET and HOM individuals. In sporadic PD, cognitive impairment has been shown to occur since early clinical stages, probably following a long non-symptomatic period of brain compensation. This means that, in our experimental model, HET individuals were expected to be less cognitively impaired than HOM patients. However, consistent with a previous study on *PINK1* mutation carriers (including part of the patients enrolled here) [12], all but one subjects from either group (HET, HOM) reported pathological scores at MoCA. Moreover, the two cognitively preserved individuals were both *PINK1* heterozygous carriers, while all *Park2* mutation carriers (3 HET, 2 HOM) showed pathological scores at MoCA. This is the first study reporting such a finding in *Park2* mutated subjects, which is apparently in contrast with previous reports [9, 10]. Nevertheless, as previously suggested, MoCA is highly sensitive in detecting cognitive deficits in PD [33], and this might account for inconsistences across studies. Additionally, the similar cognitive profile we observed in *PINK1* and *Park2* mutation carriers is congruent with recent neuroimaging studies, suggesting a similar endophenotype for the two genes [7]. After characterizing the cognitive profile of all recruited subjects, we focused on the patterns of FC, in order to explore potential substrates for their neuropsychological characteristics. We focused our analysis on five specific networks that, according to previous literature [16, 18, 35–37], have proven to be meaningful in reflecting brain connectivity abnormalities in PD. We first analyzed each network in isolation (intra-network connectivity), and then we explored their interaction (inter-network connectivity) as a function of disease severity. When considering the DMN in isolation, both genetic groups (HOM and HET) compared to controls revealed reduced connectivity in the posterior cingulate cortex, with no significant differences between them. This finding fits well with the pathological scores reported by this genetic cohort at MoCA (i.e., a measure of global cognition) irrespective of their group belonging (HOM or HET). The posterior cingulate cortex is regarded as one of the most critical nodes of the DMN, whose connectivity is disrupted proportionally with global cognition not only in patients with Alzheimer’s disease [17], but also in those with sporadic PD [18, 35]. Additionally, in patients with both diseases, reduced connectivity between the posterior cingulate cortex and the rest of the brain has been found since early clinical stages, preceding and perhaps contributing to local GM atrophy [16, 17].

Beyond the MoCA assessment, HOM patients, compared to HET individuals, performed significantly worse in tests for spatial working-memory. Consistently, HOM patients revealed a remarkable reduction of FC also in other networks (i.e., rFP, ExN), which can be more directly referred to working-memory [38]. In these same networks, HOM patients could be differentiated not only from HC but also from HET individuals. We speculate that disruption of rFP and ExN parallels the observed disability in more specific areas of cognition. Indeed, spatial working-memory deficits are often reported in sporadic PD, due to both a limited storage capacity and inability to filter out distracting information [2]. Moreover, we found a direct association between performances at Corsi test by HOM patients and the strength of FC within ExN, SN, rFP and lFP. These networks all involve the frontoparietal cortex and overlap at both the medial and lateral frontoparietal cortex [39]. Indeed, they are postulated to exert cognitive functions of control [15], and their implication in working-memory is well described [39].

Beyond the role of single networks in specific cognitive functions, interactions between them are likely to account for the appearance of complex symptoms along disease evolution, as well as for compensation mechanisms typically observed in preclinical stages of neurodegenerative dementias [19]. In a recent work by Gorges and co-authors, different patterns of FC have been reported in patients with PD according to the presence/absence of cognitive impairment [40]. Cognitively impaired patients showed reduced FC, especially within the DMN. Conversely, cognitively unimpaired patients revealed a widespread increase of FC probably reflecting “compensatory” mechanisms.

We first confirmed the negative correlation reported in literature between the DMN and other positive networks [41, 42]. In normal individuals, greater negative correlations between the DMN and fronto-parietal networks [42] have been associated with improved performance (and less ‘‘mind wandering”) on tasks requiring externally-directed attention. These negative correlations suggest that the brain may be intrinsically organized to support competitive relationships between networks involved in external attention and internally focused thoughts. When looking at group differences, we found a paradoxical positive correlation between the DMN and SN, rFP and lPF networks in HET individuals only. This might represent a compensatory mechanism of early PD stages, resulting in enhanced communication between the DMN and anterior networks. Indeed, such an effect was not observed in HOM patients. The hypothesis of compensatory processes is further supported by behavioral data showing a better performance in visual short-term working-memory in HET individuals compared to HOM patients. Compensatory mechanisms have already been described in asymptomatic *Park2* and *PINK1* mutation carriers, showing a stronger increase of cortical motor-related activity during execution of self-initiated movements. These changes were interpreted as an evidence for a large-scale reorganization of the motor system in the presymptomatic PD [6, 7].

Within the internetwork analysis, we found a strong effect which was peculiar of HOM patients, with an increase of inter-network connectivity between SN and rFP networks. This finding is of non-obvious interpretation, due to the lack of association with neuropsychological data. However, we argue that such an effect might reflect a compensation mechanism occurring at more advanced disease stages, although further longitudinal studies are needed to confirm this interpretation.

We are aware that the present study suffers from some limitations. First, the number of subjects included in the study is relatively small, due to the fact that autosomal recessive forms of PD are relatively rare. Moreover, HOM but not HET individuals nor HC were under dopaminergic therapy. This could have affected the results by artificially “normalizing” FC values within specific networks [34]. Nevertheless, we did not find any significant correlation between functional connectivity and LEDD in HOM patients. While it is of course important to acknowledge this potential confound, most of our findings should not be directly affected by it.

In conclusion, this study validates the use of RS-fMRI in spotting pathophysiological dysfunctions in ARPD and their relationship with cognitive impairment, especially within the visuo-spatial working memory. Current therapies in PD primarily target the motor symptoms, although cognitive decline is known to affect 15–20% of all patients and impact on patients’ and relatives’ quality-of-life. Altered resting-state FC in PD, reflecting clinically relevant phenomena, holds promise as a marker of disease progression. Follow-up of our cohort of mutated subjects will allow us to assess whether changes in FC can serve as a predictor for cognitive decline, especially in HET individuals. The combination of genetic and functional neuroimaging information may prove useful for monitoring individuals at risk for developing PD before the onset of cognitive symptoms, and it is critical for planning neuro-rehabilitation programs.

## Abbreviations

ARPD: autosomal recessive Parkinson’s disease
BOLD: blood-oxygen-level dependent
DMN: default mode network
ExN: executive network
FAB: Frontal Assessment Battery
FC: functional connectivity
GM: grey matter
HET: heterozygous/heterozygotes
HOM: homozygous/homozygotes
HC: healthy controls
lFP: left fronto-parietal network
MoCA: Montreal Cognitive Assessment battery
RS-fMRI: resting-state functional MRI
rFP: right fronto-parietal network
SN: salience network
